# "A Free thenar flap – A case report"

**DOI:** 10.1186/1749-799X-2-4

**Published:** 2007-03-12

**Authors:** Rajesh Garg, Boris KK Fung, Shew Ping Chow, Wing yuk Ip

**Affiliations:** 1 Department of Orthopaedic Surgery, Hand and Foot Division, Queen Mary Hospital, University Of Hong Kong, Hong Kong

## Abstract

We present a case report of a free thenar flap surgery done for a volar right hand middle finger, distal and middle phalanx degloving injury. A free thenar flap is a fasciocutaneous sensate flap supplied by a constant branch of the superficial radial artery and its variable nerve supply. It has a distinct advantage of low donor site morbidity, better cosmesis and texture of the flap. No immobilization is required postop. The donor site can be closed primiarily.

## Background

Numerous local or regional flaps have been used to cover medium to small size volar soft tissue defects of the digit. Large volar defects over the digit have presented a therapeutic challenge to the reconstructive hand surgeon. Free flaps from the feet or toes have been used to provide satisfactory coverage of these large defects, however donor site morbidity is unavoidable and the patient's acceptance is questionable.

We would like to report an alternative method to resurface a large volar defect of the finger utilizing a free thenar flap.

## Case report

**Figure 1:**
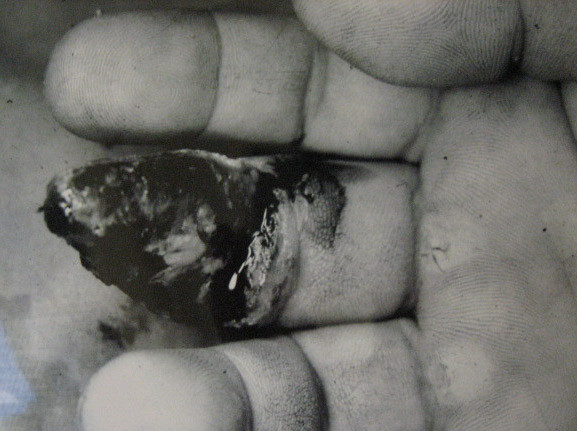
Showing the extent of injury.

A 36 year old man sustained a degloving injury to his right, middle finger (which he caught in a machine, while at work) resulting in a large volar soft tissue defect extending from the tip of the distal phalanx to the mid portion of the middle phalanx. Bone and part of the profundus tendon was exposed (Fig. 1). The tip of the distal phalanx was crushed, without any other bony injury.

A primary debridment was done on the day of injury, because the wound was contaminated with grease and grit in the emergency operation theatre. The exposed tendon and bone was covered with a collagen dressing.

When the wound was inspected on day 3, it was found to be healthy and a flap was planned to cover the exposed tissues.

We have had a lot of experience with cross finger flaps and free flaps from the toe and foot. However, they have been associated with lack of patient compliance, morbidity to the donor areas and immobilization in the case of cross finger flaps. Therefore, we planned to do a free thenar flap, based on the superficial branch of the radial artery. We had carried out cadaver dissections and found the vascular supply consistently associated with this fasciocutaneous flap. This fasciocutaneous flap would have a texture similar to the pulp tissue. The other main advantage of the free thenar flap would be its sensory supply by either of the nerves (palmar cutaneous branch of median nerve, lateral antebrachial cutaneous nerve or branch of superficial radial nerve).

**Figure 2:**
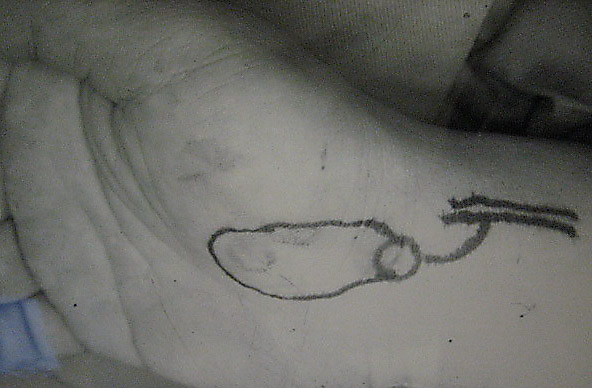
Showing the blue print of the flap area and the pedicle.

**Figure 3:**
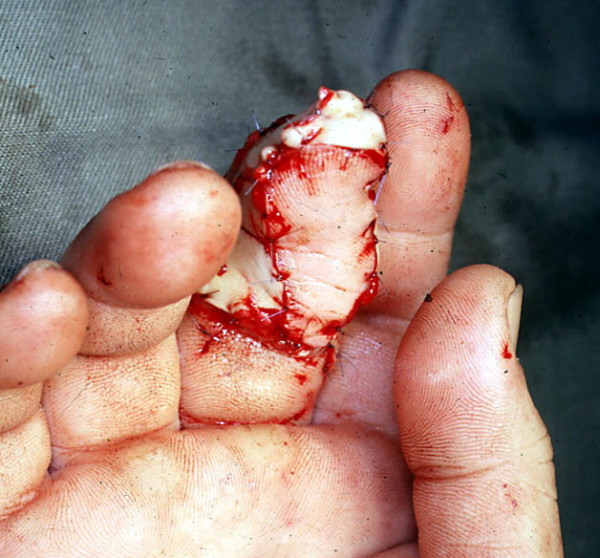
Immediate postop clinical picture showing good vascularity of the thenar flap.

On day 4, a free fasciocutaneous thenar flap was performed under regional block. A blue print of the flap is shown in the figure 2. No upper limb exsanguinations was done, which helped in identifying the thin vessels under the loupe. An upper limb tourniquet was used to minimize bleeding. A thenar flap measuring 4 × 2.5 cms was dissected with the vascular and neural pedicle (a branch of the superficial radial nerve). The tourniquet was released intra-operatively after the neuro-vascular anastomosis was completed. Blood flow was adequate (figure 3). The donor site over the palmar aspect was primarily sutured. The operation took approximately 6–7 hrs. A rigid dressing was applied to reduce post operative edema. The middle finger along with the wrist was immobilized (4 days) to reduce postoperative pain and to help in initial wound healing.

On day 9 (post-operative day 5), the digit was redressed. Both the donor and the recipient site were found healthy. Sutures were removed on day 15 (11 days after the operation). Physiotherapy was started for the middle finger and wrist, from the 4^th ^post-operative day.

**Figure 4:**
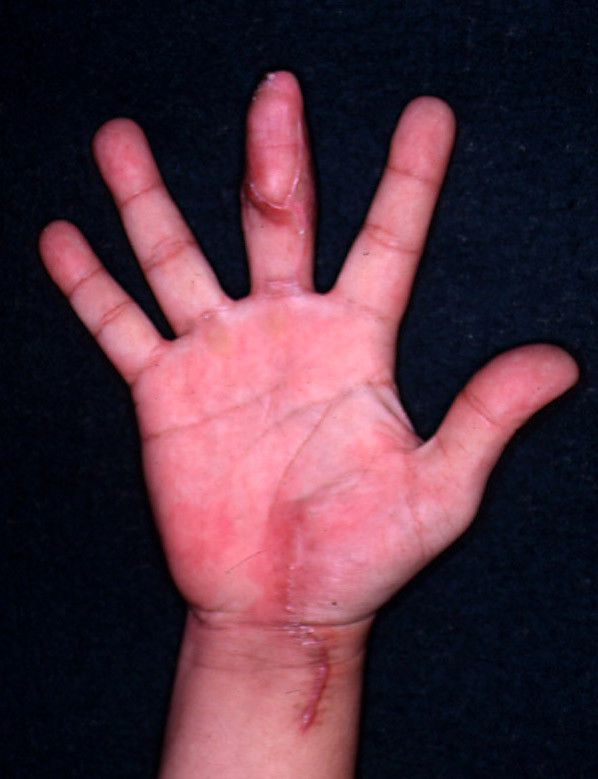
Volar aspect of the middle finger (free thenar flap 6 months postop) and also the healed donor site.

**Figure 5:**
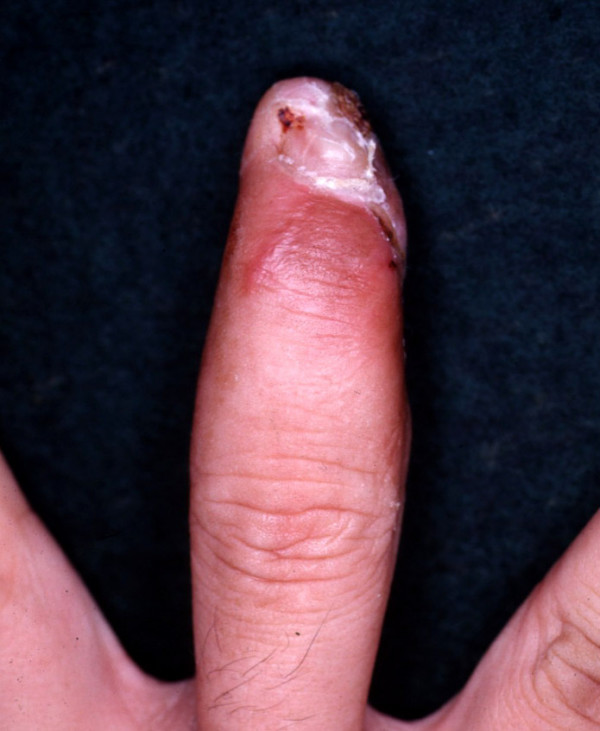
Dorsal aspect of the middle finger after free thenar flap, 6 months postop.

6 months after the injury, the patient is satisfied with the flap. He is happy about the texture of the flap which matched the other fingers

(Figure 4 and figure 5). He has 90% deep touch sensations and approximately 50% soft touch sensations. The only uncomfortable sensation he has had was transient tightness over the palmar scar which had disappeared with time.

## Discussion

Free thenar flap was first used by Kamei [1] in 1993 and later by Tamai [2] in 1996. They used it successfully on seven patients. An anatomical study of the flap was done in 1997 by Pilz and Omokawa [3].

The flap is based on the superficial branch of the radial artery. This artery was seen to be constantly present in all the cadaver dissections [4] that were done. It branches out from the radial artery 2.5 cm proximal to the scaphoid tubercle with a pedicle length of 2 cm. The average diameter of the vessel ranges from 0.8–1.4 mm. It supplies a constant skin area of 3 × 4 cm. In addition to being a fasiocutaneous flap, it is a sensory flap (supplied by the palmar cutaneous branch of median nerve, lateral ante brachial cutaneous nerve or branch of superficial radial nerve) with a texture that closely matches the pulp tissue. If the width of the flap is less than 2 cm, the donor site can be closed primarily.

We advocate this flap as it is a fasciocutaneous sensate flap, locally available from the same injured hand, thereby decreasing donor site morbidity and a preferred flap by patients in comparison with cross finger flaps or flaps from the toe or foot. The flap has adequate subcutaneous tissue to give it the texture of pulp and also the genotypic appearance of the lost cover. It has a constant vascular pedicle (superficial branch of the radial artery). The donor site of the flap (thenar eminence) can be closed primarily, if the size is less then 2 cms, with minimal scarring. No postoperative immobilization is required unlike cross finger flaps.

Like other free flaps, this thenar free flap also has some risk of failure of flap due to loss of circulation postoperatively. The patient may also complain of pain at the site of detachment of the donor nerve, if the nerve was not carefully dissected and buried, due to neuroma formation. It can sometimes be very tedious to identify the nerve in the flap.

We started this project by reviewing the literature, dissecting cadavers and checking the consistency of the neurovascular pedicle. Our first case was successful and we intend to use this flap to cover medium to large digital soft tissue defects when conventional means are not feasible.
